# Role of C-fibers in pain and morphine induced analgesia/hyperalgesia in rats

**Published:** 2014

**Authors:** Zahra Alizadeh, Masoud Fereidoni, Morteza Behnam-Rassouli, Shirin Hosseini

**Affiliations:** Department of Biology, Faculty of Sciences, Ferdowsi University of Mashhad, Mashhad, Iran

**Keywords:** C-Fibers, Pain, Analgesia, Hyperalgesia, Morphine

## Abstract

**Background:**

Usual dosage of morphine (10 mg/kg) induces analgesia and ultra-low dose (ULD) of morphine (1 µg/kg); hyperalgesia, and C-fibers are also bearing µ-opioid receptors; here the importance of C-fibers on pain and morphine induced analgesia/hyperalgesia is questioned and investigated using pain evaluation methods and infant capsaicin treating for C-fibers lesioning.

**Methods:**

Wistar male rats (200-250 grams) were assigned to three categories i.e. control, sham (receiving neonatal capsaicin vehicle) and c-lesion (receiving neonatal capsaicin), each one with three groups (n=7). They were injected intraperitoneally with single dosage of saline, 10 mg/kg or 1 µg/kg morphine, respectively. Thermal pain threshold was evaluated using the tail flick test before and 30 minutes after the injections. Chemical pain was assessed using the formalin test (FT) 30 minutes after the administrations.

**Results:**

Results indicated that thermal (P < 0.001) and chemical pains in both neurogenic and inflammatory phases of FT (P < 0.05) were reduced in C-lesion animals. In the C-normal and C-lesion animals, 10 mg/kg morphine exerted analgesia both in thermal (P < 0.001) and two phases of FT (P < 0.01), but it was more potent in C-lesion animals (P < 0.05). Although ULD of morphine in C-normal animals produced hyperalgesic effect in thermal and chemical pains (P < 0.001), in C-lesion animals, it produced analgesia (P < 0.05) at the neurogenic phase of FT.

**Conclusion:**

Results can raise the C-fibers involvement for a significant portion of nociceptive transmission, because C-lesioning potentiated morphine induced analgesia and eliminated ULD of morphine induced hyperalgesia. Therefore C and Aδ fibers can be involved in morphine analgesia; while, just C-fibers are possibly responsible for only presynaptically hyperalgesic/excitatory action of ULD in morphine.

## Introduction

Nociception is a part of bodily defense mechanism. It alarms the animals for damaged or potentially injured tissues and pushing them to act and remove nociception-inducing factors.^1^ Human beings are constantly trying to seek pain remedies. Hence; lot of efforts are being put to learn the basic concepts of pain and nociception mechanisms, including its sensation, transduction and perception. One of the most glorious discoveries in this regard was the brain circuits of pain modulatory system.^2^ Terminals of C-fibers or non-myelination nociceptive afferent, at the level of spinal cord dorsal horn, are one of the targets of this modulatory system with the action of opioidergic system.^3^ Many studies indicate that opioids are very effective analgesic compounds.^4^ Opioids, such as morphine that are used clinically to alleviate severe to moderate pains in cases of cancer or after surgeries, seem to inhibit nociceptive signals at the level of synapses within the dorsal horn of the spinal cord using pre and postsynaptic inhibitory mechanisms. They can diminish presynaptic Ca2^+^ influx leading to a decrease in neurotransmitter release. Moreover, opioids can activate G protein coupled K^+^ channels (GIRK) and hyperpolarize the postsynaptic neurons.^5, 6^ Opioids receptors (ORs) are belonging to a large family of G protein coupled receptors (GPCRs). So analgesic effect of opioids is due to the coupling of ORs to the inhibitory G proteins (Gi/Go) especially on nociceptive afferent neurons of C-fibers. This inhibits cAMP production and then either inhibits voltage operated Ca^2+^ channels (VOC) or activates K^+^ channels to hyperpolarize the cell.^4, 7, 8^

In the middle of 1970s, there were some reports, which showed an excitatory activity for opioids.^9^ Studies on cultured neurons of dorsal root ganglion revealed that opioids at very low concentrations, below the usual doses, which have neuronal inhibitory action, exert an excitatory effect at nanomolar concentrations of opioid agonist that increase the action potential duration time while micromolar concentrations have opposing to. A model of opioid's receptors bimodal action explains these dual effects of opioids. Based on this model, ultra-low doses of opioid agonists activate an opioid receptor; that activates stimulatory G protein (Gs) and adenylyl cyclase, which in turn elevates the intracellular cAMP and neuronal excitability. These effects lead to a hyperalgesia which can be blocked using ultra-low doses of naloxone or naltrexone; while; usual doses of opioid's agonist exert their analgesic effects when their receptors couples to inhibitory G protein (Gi/Go), recent effect can be reversed by the higher doses of opioid antagonists.^10^ However, growing body of evidence has demonstrated that these receptors can physically interact with a variety of accessory proteins, showing that signal transduction of the opioid receptors is not restricted to heterotrimeric G protein activation trough a linear signaling.^11^ The opioid receptor interacting proteins (ORIPs) probably play a role in response to opioid agonists. Based on this interactions, the µOR signalplex are hypothesized to be important for µOR signaling which explain the cellular mechanisms of OR signaling in brain and may be critical for determining the physiological basis of opioid tolerance and addiction.^12^ This can be important clinically, because usual dose of opioids as painkiller can potentially express such hyperalgesic effects on the patients,^13, 14^ easily it can be imagine that opioid dosage will be reached to such mentioned ultra low dose pharmacokinetically. Thus, any insight in to the mechanism of opioids-ultra low dose action on pain by investigation can help us to find probable ways to clinically use opioids without their restrictions. A wide range of studies have confirmed that opioid receptors are excited on neurons of C-fibers, Aδ-fibers, visceral fibers containing vanilloids receptors type 1 (TRPV1), B4 isolectin, substance P (SP) and calcitonin gene-related peptide (CGRP) expressing neurons, sympathetic neurons and immune cells.^4, 5^ It was shown that density of opioid µ receptors on matured rat spinal cord which is treated by capsaicin at their infant stage diminished by sixty percent.^15^ Regard to C-fibers role in the transmission of noxious stimulus and opioid µ receptor existence on them, the present study is designed to question and compare the role of C-fibers on pain sensation and the dual effects of morphine as an opioid agonist, including analgesia at usual dose and hyperalgesia at the ultra-low dose. To address the question, C-fiber lessening performed using common method of infant capsaicin (50 mg/kg, i.p.) treatment,^16–18^ along with methods of thermal and chemical pain assessments were used in this study.

## Materials and Methods

### Animals

Male Wistar rats weighing 200-250 g were used for the study, with seven rats per group. The animals were housed in the plexy glass cages and kept at 22±2°C, 12 h Dark/12 h Light (lights on at 07:00) and given food and tap water *ad libitum*. For more environmental adaptation, the animals were placed on the laboratory one week before the onset of the experiment; they were taken out of their cages merely for experimental performances. The experimental protocol complied with the National Institutes of Health Guide for Care and Use of Laboratory Animals (NIH Publication No. 85-23, revised 1985). Rats were divided into three main categories, 1) C-lesion; infants in this category were treated with 5% capsaicin (Sigma, Germany) at a dose of 50 mg/kg intraperitoneally (i.p.) 24-48 h after birth.^19–22^ After maturation, male animals weighing 200-250 g were divided into three groups, the first one was treated with saline, second with analgesic/usual dose of morphine (10 mg/kg, i.p),^10^ and third with hyperalgesic/ultra low dose of morphine (1 µg/kg, i.p).^10^ 2) Sham; infants were treated 24-48 h after birth only by i.p. injection of capsaicin vehicle including saline, tween 80, and ethanol in the ratio of 8:1:1. After maturation, they were divided into subgroups similar to the first category. 3) Control; infants of this group remained intact, after maturation, they just received saline intraperitoneally. All i.p. injections were performed at a volume ratio of 1 cc/kg and the experimenter was blind to drugs treatments.

### Chemosensitivity corneal test

To confirm the C-fibers elimination, due to the capsaicin action in the infants, animals were exposed to the chemosensitivity corneal test after maturation prior to any other treatment. Briefly, one drop of 1% ammonium hydroxide was placed over the rat eye, and then the wipe number was recorded for 10 sec imediately after dropping. Significant alleviation in the wipe number can be interpreted as C-lesion in the infant capsaicin treated animals in contrast to sham and control animals; whereas, the responses to chemical stimulants were usually mediated by C-fibers.^22^

### Tail flick test

Thermal pain threshold was evaluated using tail flick test based on the D'Amour and Smith method.^20^ Briefly, animal withdraws its’ tail when is exposed to the concentrated burning light on the middle one third of the tail, after a while (Latency Time). Light intensity of the tail flick apparatus (Sparco, Iran) was adjusted to make a 4 to 5 second latency time in the intact animal. A cut-off time of 15 second was considered to prevent any possible tissue damage. Latency time was recorded thrice with one-minute interval for each set of the tail flick test; the mean was considered as a thermal pain threshold (tail flick latency) which was measured before and 30 minutes after drug administration, this time period is considered for complete systemic distribution of the drug in the body of adult rats.^23^ The maximum possible effect percentage (MPE%) was calculated using the following formula:^24^

$$\text{MPE}\%=\frac{\text{Post Drug Latency}-\text{Pre Drug Latency}}{\text{Cut-Off time}-\text{Pre Drug Latency}}$$

### Formalin test

As explained above, 30 minute after the drug administrations, Chemical pain evaluation was carried out based on Dubuisson and Dennis method.^25^ In brief; 0.05 ml of 2.5% formalin solution was injected at the sub plantar region (s.p.) of right hind paw and by 15-second intervals, the animal behavior scores against pain sensation were recorded for duration of one hour. As a score of zero indicates “no pain”, the animal is walking easily and the paw is placed on the floor comfortably; score 1 is given when the animal is restraining from any full paw contact to the floor; score 2 is given when the paw is fully taken above; and score 3 is given for the time when the animal is biting, shaking or licking the paw. The mean of scores was calculated in the five-minute time intervals.^25, 26^

### Statistical analysis

The data were expressed as mean ± SEM. Repeated measure ANOVA was performed for each group of formalin test. One-way ANOVA and T_tukey_ post hoc test were applied using GraphPad Prism 5 software to evaluate the treatments effectiveness and to compare the means between the groups. P<0.05 was considered as significant difference. Graphs were prepared using the Microsoft Excel 2007 software.

## Results

**Figure 1:**
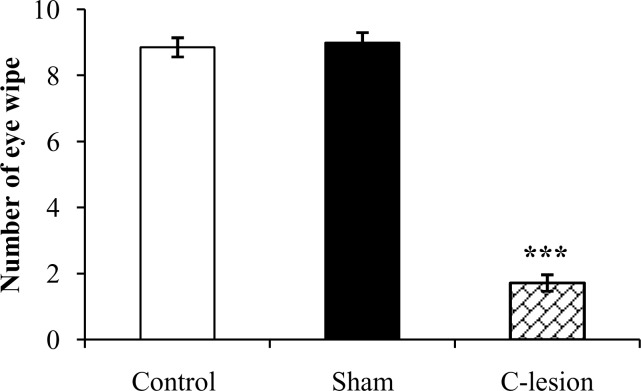
Eye wipe comparison between control, infant capsaicin vehicle treated (sham), and infant capsaicin treated animals (C-lesion) after dropping of ammonium hydroxide in the eye. In group infant capsaicin treating, eye wipes number is diminished in contrast to control and sham, so C-normal is considered for control and sham groups animals and C-lesion for animals of group infant capsaicin treating. Data are shown as mean ± SEM (***P<0.001 in contrast to control and sham) (n=7).

At first, it was required to clarify the effects of infant capsaicin treatment and C-fiber lesioning on thermal and chemical pain sensation in the mature animal using tail flick and formalin test respectively, then the results of the mentioned clarification making it possible to interpret the effects of C-fiber lesioning on the morphine induced analgesia and hyperalgesia. In contrast to control and sham (C-normal) animals results for the test of chemosensitivity of the cornea before any other tests had shown that after dropping of the ammonium hydroxide within the eye, wipes were diminished in infant capsaicin treated animal [F(2, 18) = 263.9, P<0.001] (Fig. 1). These results provided a confirmation for C-fiber elimination happening in infant capsaicin treated (C-lesion) animals.

### Tail flick test

**Figure 2:**
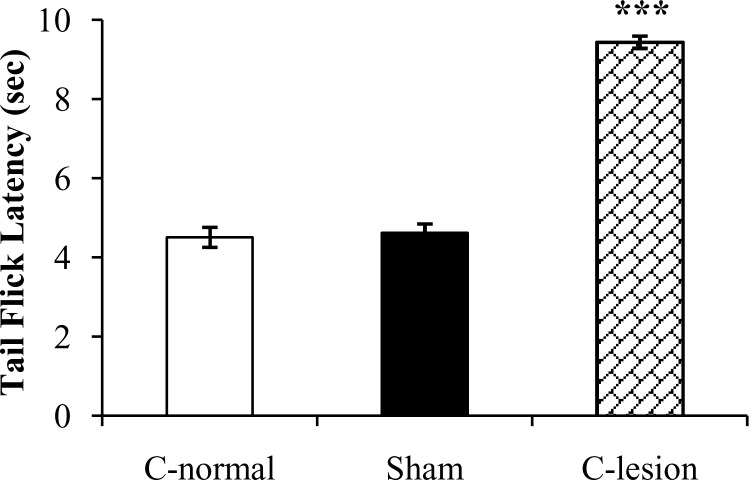
Thermal pain threshold comparison between sham, control (C-normal) and C-lesion (infant capsaicin treated) animals. Infant's capsaicin treating increased thermal pain threshold thus induced thermal analgesia. There is no difference between C-normal animals, control and sham. Data are shown as mean ± SEM (*** P < 0.001 in contrast to control and sham) (n=7).

The results have shown that the thermal pain threshold diminished after maturation in C-lesion animals in contrast to sham and control (C-normal) animals [F(2, 18) = 563.9, P<0.001] (Fig. 2). There was no difference between sham and C-normal; therefore, neither infant capsaicin vehicle treatment nor saline treatment after maturation had any effect on pain threshold. Instead, infant capsaicin treatment exerts a significant elevation in the thermal pain threshold (analgesia) after maturation.

**Figure 3:**
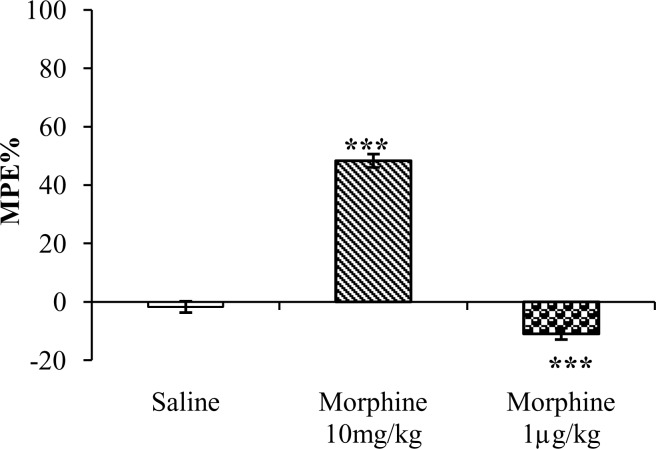
Comparison of the effects of i.p. morphine at doses of 10 mg/kg and 1 µg/kg in C-normal animals. Morphine 10 mg/kg showed 50% analgesia while morphine 1 µg/kg induced a 10% hyperalgesia in contrast to control (saline). Data are shown as mean ± SEM (*** P<0.001 in contrast to control) (n=7).

Morphine (10 mg/kg, i.p.) in C-normal animals induced analgesia with MPE% close to 50% but morphine (1 µg/kg, i.p.) induced hyperalgesia with MPE% next to 10% [F(2, 18) = 357.6, P<0.001] (Fig. 3).

**Figure 4:**
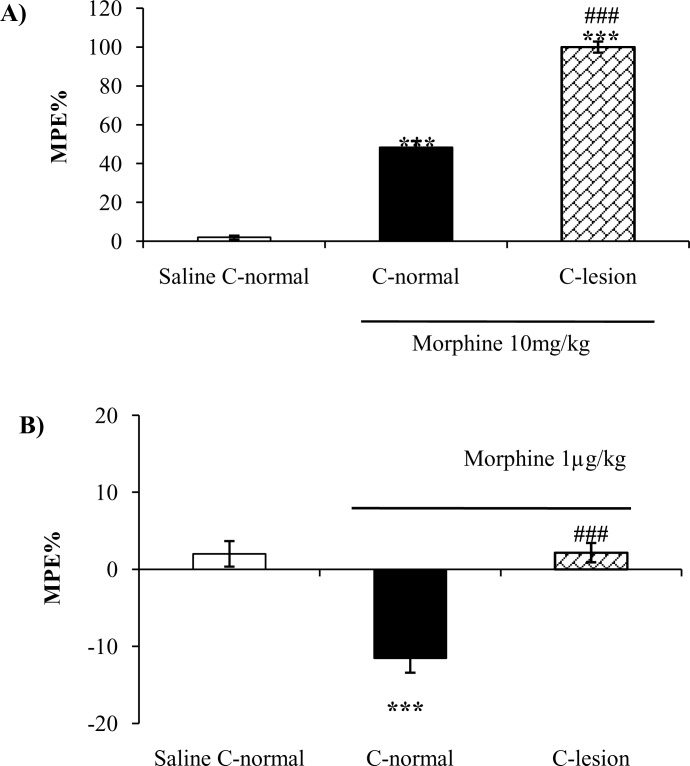
Comparison of the analgesic and hyperalgesic effects of i.p. morphine (10 mg/kg and 1 µg/kg) between C-normal and C-lesion animals. (A) Morphine 10 mg/kg induced 50% analgesia in C-normal animals while in C-lesion animals the analgesia induced by the same dose of morphine reached almost to 100%. Data are shown as mean ± SEM (***P<0.001 in contrast to i.p. saline treatment in C-normal animals, ###P<0.001 in contrast to i.p. morphine 10 mg/kg treatment in C-normal animals) (n=7). (B) Morphine 1 µg/kg induced a 10% hyperalgesia in C-normal animals while in C-lesion animals, the hyperalgesia is reversed. Data are shown as mean ± SEM (***P<0.001 in contrast to i.p. saline treatment in C-normal animals, ###P<0.001 in contrast to i.p. morphine 1 µg/kg treatment in C-normal animals) (n=7).

In contrast to sham and control (C-normal), morphine (10 mg/kg, i.p.) analgesia became more potent in C-lesion animals with MPE% of 100% [F(2, 18) = 414.7, P<0.001] (Fig. 4A). Results also showed that morphine (1 µg/kg, i.p.) hyperalgesia was reversed significantly in the C-lesion animal in contrast to C-normal animal [F(2, 18) = 7.663, P<0.001] (Fig. 4B).

### Formalin test

**Figure 5:**
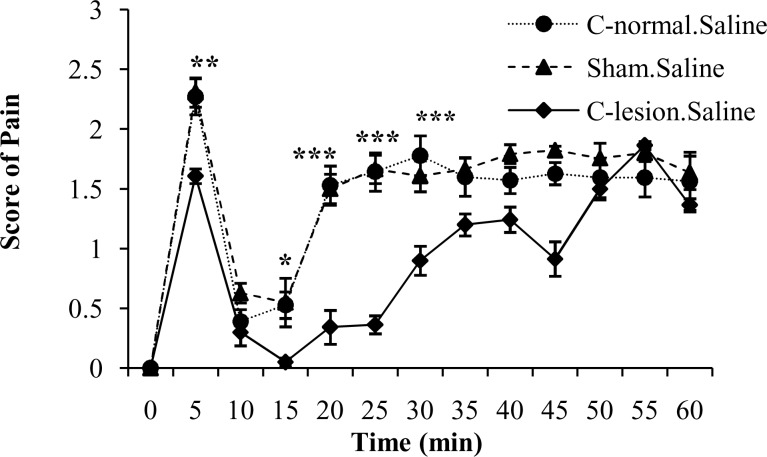
Comparison of the results of formalin test between control, sham (C-normal) and C-lesion (infant capsaicin treated) animals, all groups were treated by saline after maturation. Pain induced by formalin decreased in the most of the time of both neurogenic and inflammatory phases of formalin test in the C-lesion animals respect to C-normal. Data are shown as mean ± SEM (*P<0.05, **P<0.01, ***P<0.001 in contrast to C-normal animals) (n=7).

Chemical pain sensations were the same for sham and control (C-normal) animals and consist of two main phases. The first phase, called neurogenic phase, proceeds during the initial 10 minutes. The second phase proceeds 15 or 20 minutes after the formalin s.p. injection and lasts 40 minutes or more. This phase is also called inflammatory phase due to the inflammatory mediators involvement in the pain sensation and transduction.^22^ The comparison between sham and control groups showed no differences, as infant capsaicin vehicle treatment had no significant effect on chemical pain sensation in formalin test. The results indicated that the pain induced by formalin was alleviated both in the neurogenic and inflammatory phases in C-lesion animals [F(38, 234) = 25.39, P<0.05] (Fig. 5), in contrast to C-normal animals.

**Figure 6:**
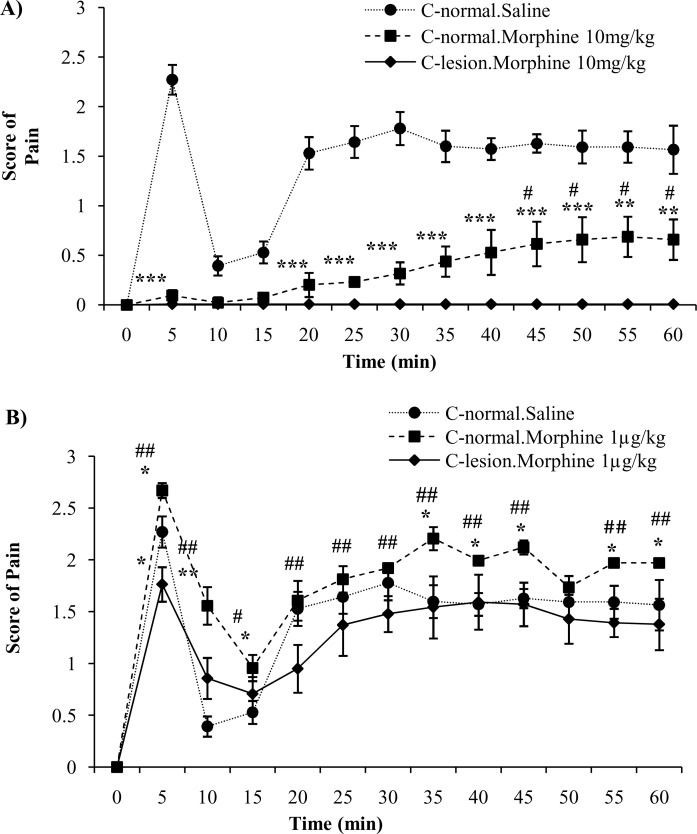
Comparison of the analgesic and hyperalgesic effects of i.p. morphine (10 mg/kg and 1 µg/kg) between C-normal and C-lesion animals in formalin test. (A) Morphine 10 mg/kg alleviated the chemical pain sensation induced by formalin both in the neurogenic and inflammatory phases in C-normal animals and showed the analgesic effect; while, the same dose of morphine alleviated the pain sensation in C-lesion animals even more than that happened in C-normal animals, especially in inflammatory phase. Data are shown as mean ± SEM (**P<0.01, ***P<0.001 in contrast to C-normal. Saline, #P<0.05 in contrast to C-lesion. Morphine 10 mg/kg) (n=7). (B) Morphine 1 µg/kg increased the chemical pain sensation induced by formalin both in the neurogenic and inflammatory phases in C-normal animals and showed the hyperalgesic effect; while, the same dose of morphine interestingly produced an analgesic effect in the neurogenic phase and no special effect in the inflammatory phase of formalin test in the C-lesion animals, so at least it could be inferred that in C-lesion animals, previous hyperalgesic dose of morphine was not able to induce hyperalgesia. Data are shown as mean ± SEM (*P<0.05, **P<0.01 in contrast to C-normal. saline, #P<0.05, ##P<0.01 in contrast to C-lesion. Morphine 1 µg/kg) (n=7).

Morphine (10 mg/kg, i.p.) in C-normal animals diminished the pain in both formalin test phases [F(38, 234) = 28.76, P<0.01] (Fig. 6A), this effect was more potent in C-lesion animals as they did not suffer from almost any pain at all (P<0.05) (Fig. 6A). Morphine (1 µg/kg, i.p.) in C-normal animals elevated pain sensation in both neurogenic and inflammatory phases of formalin test (P<0.05); while, this hyperalgesic morphine interestingly showed an analgesic effect in the neurogenic phase [F(38, 234) = 14.01, P<0.05] and had no hyperalgesic effect in inflammatory phase of formalin test in C-lesion animals (Fig. 6B).

## Discussion

The role of C-fibers on thermal and chemical pain sensation, analgesic morphine (10 mg/kg, i.p.) and hyperalgesic morphine (1 µg/kg, i.p.)^10^ were studied using capsaicin treatment in infants for C-fibers elimination. The results confirmed that a high i.p. capsaicin dose of 50 mg/kg, in one-to-two-day infants as a routine method of C-lesioning in the investigations can degenerate and remove most of C-fibers because of its neurotoxic effect.^16–22, 26^ Likewise, the results showed that thermal pain threshold in tail flick test was elevated in C-lesion animals; however, analgesia did not reach to the maximum. The same circumstance happened both in the first (neurogenic) and second (inflammatory) phases of formalin test for chemical pain. It was revealed that noxious thermal and chemical stimuli could lead to the release of glutamate, SP and CGRP from the central nociceptive afferent terminals during nociceptive signal transmission, which excited the protective reflexes or pain-related behavior. Withdrawal reflex is one of them that separate the stimulated organ from the stimulant, like what can be seen in the tail flick against thermal radiation, which focused on the tail.^27, 28^ Because of C-fiber neurons sensitivity to noxious thermal and chemical stimulants and release of SP and CGRP peptides by C-fibers terminals,^29, 30^ it is expected that C-fibers are important for thermal and chemical pain transmission. This assumption is augmented by our results, which showed that thermal and chemical pain sensations were diminished in C-lesion animals. However, it was not diminished completely, but why? It might be the case that a part of the pain induced in these tests has been conducted by another type of the thermal and chemical stimulants’ sensitive fibers such as Aδ. They release glutamate, as an excitatory neurotransmitter, at their central terminals on the dorsal horn of spinal cord.^31, 32^ Peripheral and central release of inflammatory mediator is believed to be the cause of pain sensation in the formalin test second phase;^33^ as the results showed, pain in this phase was alleviated in the C-lesion animals. This can address the C-fibers as an important factor in inflammatory mediator release. Although, it is impossible to ignore the direct or indirect roles of the other fibers like as Aδ, in the central and peripheral release of inflammatory mediator, because our results showed that a part of pain sensation remained in the C-lesion animals in the second phase of formalin test. This warrants further studies to explain their role.

The results were shown that morphine (10 mg/kg, i.p.) analgesia was potentiated significantly both in the thermal and chemical nociceptions. Morphine exerts its inhibitory effect on pain transmission via opioid coupling to inhibitory G proteins pre-and postsynaptically.^34–36^ It was shown that the µ opioid receptors density was diminished by 60% in spinal cord of adult rats, which underwent infant capsaicin treatment.^15^ Thus, a significant portion of µ opioid receptors are still remained perhaps on the Aδ fibers neuron in C-lesion animals,^37^ those that may be responsible for nociception even after C-lesioning in our experiments. Therefore, analgesic morphine can possibly block such remaining opioid receptors on Aδ fibers and, hence, lead to the production of potentiated analgesia, even sometimes 100%, in C-lesion animal. However, recent studies have shown an enhancing of µ opioid receptors function by physical heterodimeric association between µ and d opioid receptors, as d receptor antagonists enhance morphine-mediated intrathecal analgesia.^38^ Thus, exciting of such possible interactions also needs to be considered for potentiating of morphine analgesia in c-lesion animals for further investigations.

It is already mentioned that Aδ fibers may play a role in the inflammatory phase of formalin test; this assumption can be supported by the results, which showed analgesic morphine reduced pain in C-lesion animals more than C-normal animals in the second phase of formalin test. Hence, it can be suggested that, the Aδ fiber neurons are involved in peripheral and central inflammatory mediators release during inflammatory nociception.

The results revealed that ultra-low dose of morphine (1 µg/kg, i.p.) reduced thermal pain threshold and increased chemical pain sensation in C-normal animals, thus it was producing hyperalgesia. As it was mentioned in the introduction, hyperalgesia induced by morphine-ultra low dose is mediated via G protein coupled receptors which couple the stimulatory G protein, activate adenylyl cyclase, and elevate cAMP, this can lead finally to neuronal excitability and neurotransmitters release elevation from dorsal root ganglion (DRG) neurons.^8, 10^ It can be expected that hyperalgesia induced by morphine-ultra low dose might be mediated mostly through C-fibers neuron, because of more opioid receptor's distribution on them in contrast to the other fibers such as Aδ.^39, 40^ Our results also support this idea because ultra-low dose of morphine was unable to make hyperalgesia during tail flick test in C-lesion animals. This piece of evidence also suggests that hyperalgesia induced by morphine-ultra low dose is exerted not only through C-fibers but also it is performed presynaptically. This can be different for morphine analgesia, because as discussed before, both the C and Aδ fiber neurons possibly, and both the pre and postsynaptic neurons probably,^3, 41^ contribute to the morphine inhibitory action on pain. Ultra low dose of morphine in C-normal animals showed hyperalgesic effects in the neurogenic and inflammatory phases of formalin test. while in C-lesion animals, ultra low dose of morphine interestingly produced analgesia in the neurogenic phase and for the inflammatory phase, but it could not produce hyperalgesia. These results can be interpreted such that hyperalgesic effects of ultra low dose of morphine for chemical pain is also conveyed through the C-fibers, as C-fibers elimination reduced hyperalgesia induced by morphine-ultra low dose in two formalin test phases, even ultra low dose of morphine could produce analgesia, possibly through the remaining Aδ fibers or may be postsynaptically after the elimination of C-fibers. It is also revealed that cholecystokinin (CCK) is upregulated in the rostral ventromedial medulla (RVM) during persistent opioid exposure. CCK is both antiopioid and pronociceptive via the activation of descending pain facilitation mechanisms from the RVM which enhancing nociceptive transmission at the level of spinal cord and promoting hyperalgesia.^42^ If it is hypothesized that acute exposure to morphine ultra low dose also arises such descending pain facilitation pathways which needs to be investigated, and if it suppose that this effect is happening at least partially through the spinal cords terminals c-fibers, then c-fibers elimination possibly alleviates the pain facilitating effect of descending pathways induced by morphine ultra low dose treatment. However These considerations also call for further research.

## Conclusion

In conclusion, these assumptions could arise from the present study, C-fibers are very important to transmit thermal and chemical pain as their elimination can alleviate the thermal, chemical and inflammatory nociception. Morphine analgesia might happen both via C and Aδ fibers neuron. The elimination of C-fibers removes a potential source of nociceptive signals. Therefore, the morphine analgesic effect in C-lesion animals may be exerted both presynaptically through Aδ fibers and postsynaptically on spinal cord projection neurons. Ultra low dose of morphine may exert its excitatory effect just via C-fibers neuron as their elimination lead to revealing even an analgesia for morphine-ultra low dose, making it possible to suppose only pre synaptic excitatory mechanisms (as mentioned before) for hyperalgesic action of morphine-ultra low dose and no role for Aδ fibers neuron in this state. Further research to elucidate these pathways is required.
